# Modulation of chromatin structure by the FACT histone chaperone complex regulates HIV-1 integration

**DOI:** 10.1186/s12977-017-0363-4

**Published:** 2017-07-28

**Authors:** Julien Matysiak, Paul Lesbats, Eric Mauro, Delphine Lapaillerie, Jean-William Dupuy, Angelica P. Lopez, Mohamed Salah Benleulmi, Christina Calmels, Marie-Line Andreola, Marc Ruff, Manuel Llano, Olivier Delelis, Marc Lavigne, Vincent Parissi

**Affiliations:** 1 Fundamental Microbiology and Pathogenicity Laboratory, UMR 5234 CNRS, University of Bordeaux, SFR TransBioMed, 146 rue Léo Saignat, 33076 Bordeaux Cedex, France; 2 Centre Génomique fonctionnelle Bordeaux, Plateforme Proteome, Université de Bordeaux, Bordeaux, France; 3 Department of Biological Sciences, University of Texas at El Paso, El Paso, TX USA; 4 Département de Biologie Structurale Intégrative, UDS, U596 INSERM, UMR7104 CNRS, IGBMC (Institut de Génétique et de Biologie Moléculaire et Cellulaire), Illkirch-Graffenstaden, France; 5 LBPA, UMR8113, CNRS, ENS-Cachan, Cachan, France; 6 Department of Virology, UMR 3569, CNRS, Institut Pasteur, Paris, France; 7 Institut Cochin-INSERM U1016-CNRS UMR8104, Université Paris Descartes, Paris, France; 8 International Associated Laboratory (LIA) of Microbiology and Immunology, CNRS/University de Bordeaux/Heinrich Pette Institute-Leibniz Institute for Experimental Virology, Bordeaux, France; 9 Viral DNA Integration and Chromatin Dynamics Network (DyNAVir), Paris, France

**Keywords:** Retroviral integration, HIV-1, Integrase, Chromatin, Nucleosome, FACT

## Abstract

**Background:**

Insertion of retroviral genome DNA occurs in the chromatin of the host cell. This step is modulated by chromatin structure as nucleosomes compaction was shown to prevent HIV-1 integration and chromatin remodeling has been reported to affect integration efficiency. LEDGF/p75-mediated targeting of the integration complex toward RNA polymerase II (polII) transcribed regions ensures optimal access to dynamic regions that are suitable for integration. Consequently, we have investigated the involvement of polII-associated factors in the regulation of HIV-1 integration.

**Results:**

Using a pull down approach coupled with mass spectrometry, we have selected the FACT (FAcilitates Chromatin Transcription) complex as a new potential cofactor of HIV-1 integration. FACT is a histone chaperone complex associated with the polII transcription machinery and recently shown to bind LEDGF/p75. We report here that a tripartite complex can be formed between HIV-1 integrase, LEDGF/p75 and FACT in vitro and in cells. Biochemical analyzes show that FACT-dependent nucleosome disassembly promotes HIV-1 integration into chromatinized templates, and generates highly favored nucleosomal structures in vitro. This effect was found to be amplified by LEDGF/p75. Promotion of this FACT-mediated chromatin remodeling in cells both increases chromatin accessibility and stimulates HIV-1 infectivity and integration.

**Conclusions:**

Altogether, our data indicate that FACT regulates HIV-1 integration by inducing local nucleosomes dissociation that modulates the functional association between the incoming intasome and the targeted nucleosome.

**Electronic supplementary material:**

The online version of this article (doi:10.1186/s12977-017-0363-4) contains supplementary material, which is available to authorized users.

## Background

Integration of the retroviral genome into the host chromosomes, catalyzed by the integrase protein (IN), is a prerequisite for viral replication (for a recent review on integration see [1]). This process is regulated by cellular factors at several stages including nuclear import of the preintegration complex (PIC) and association with chromatin loci [2, 3]. Integration appears to occur preferentially into nucleosomal target DNA both in vitro and in infected cells but the chromatin structures and regions targeted by the integration complexes depend on the virus [3–8]. This preferential integration into nucleosomes was assumed to be due to the preference of IN for bent DNA, which can be found at the surface of the nucleosome [9]. This was confirmed by determining the complex formed between the foamy virus (PFV) intasome capture complex and the human nucleosome, showing a close association between the retroviral IN and highly bent target DNA at the nucleosome surface [7]. Recent works confirmed that nucleosomes and DNA bendability are major determinants of the HIV-1 IN selectivity [3, 9–11]. Additionally, the functional association between retroviral intasomes and chromatin has been shown to be modulated both by chromatin and intasome structures, thereby governing their preference for specific target DNA flexibility and nucleosome density both in vitro and in vivo depending on the retroviral genus [8]. Consequently, this leads to a specific and distinct requirement for chromatin structures depending on the viral integration machinery. Indeed, previous works showed that, while Avian Sarkoma Leukosis Virus (ASLV) and HIV-1 integration was preferred in regions of the chromatin with low nucleosomes density both in vitro and in vivo, PFV and Murine Leukemia Virus (MLV) integration accommodate more easily different chromatin structures with a significant preference for regions of high nucleosomes density [8].

In the cell, these suitable chromatin loci can be reached thanks to specific interactions between retroviral intasomes and cellular targeting cofactors such as LEDGF/p75, CPSF6 and BET proteins (for reviews about integration selectivity see [3, 12, 13]). While previous studies have shown that mononucleosomes are preferential substrates for retroviral integration in vitro, it has also been reported that the physiological full-site HIV-1 integration of both viral DNA ends into polynucleosomal compacted chromatin may require coupling with local additional remodeling activity [14, 15]. Interestingly, HIV-1 integration is promoted in the chromatin regions highly transcribed by the RNA polymerase II (PolII) machinery, where the nucleosomes are highly dynamic [8, 16]. Moreover, the structure of the complex formed between the PFV strand transfer complex and the human nucleosome indicates close integrase/histones interactions that allow the target DNA to reach a suitable degree of bending for integration [7]. Taken together these data suggest that cellular chromatin remodeling activities, especially those found in the vicinity of the integration sites, may control the efficiency of retroviral integration by modulating the number of intasome/nucleosome contacts. We have investigated this issue by selecting cellular cofactors of HIV-1 integration associated with chromatin. In this work, we report the identification and functional characterization of the FACT (FAcilitate Chromatin Transcription) complex as a modulator of HIV-1 integration.

**Fig. 1:**
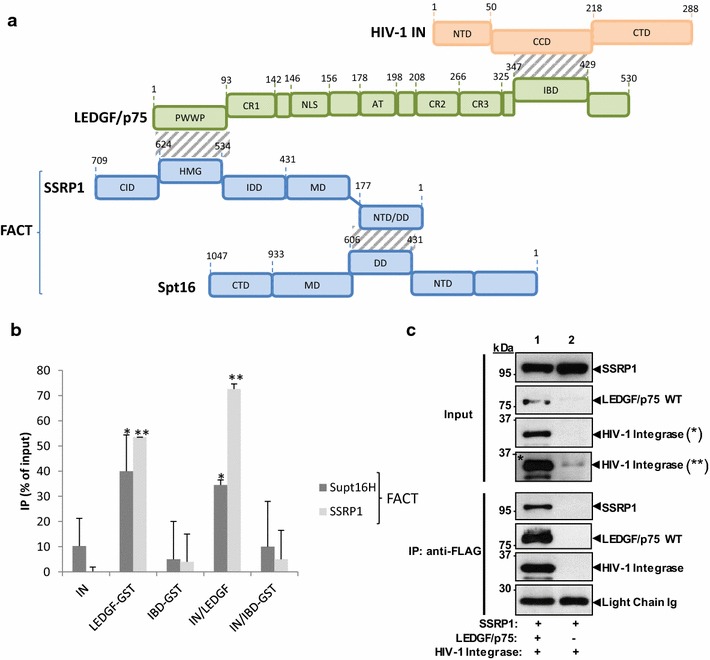
Interaction between HIV-1 IN, LEDGF/p75 and FACT complex. A Schematic representation of the previously reported interactions between HIV-1 IN, LEDGF/p75 and FACT complex is shown in **a**. IN/FACT, IN/LEDGF, LEDGF/FACT, IN/LEDGF/FACT and IN/IBD/FACT interactions were analyzed by co-immunoprecipitation using recombinant cofactors and polyclonal anti-HIV-1 IN antibodies. IBD/FACT interactions were analyzed by GST-pull down using IBD-GST and FACT recombinant proteins. The interactions were monitored either direct gel staining using colloidal blue or western blot using the corresponding antibodies and quantified by Image J software (see quantification in **b** and representative experiments in Additional file 3: Figure S3). All values are shown as the mean ± standard deviation (*error bars*) of at least three independent sets of experiments. The p-values were calculated by Student’s *t* test and are shown as *p < 0.05 and **p < 0.005 to represent the probability of obtaining significant differences compared with the data obtained with the negative background obtained with the beads alone. Cellular interaction between SSRP1, LEDGF/p75 and IN was checked by immunoprecipitation with an anti-FLAG antibodies in cells lysates obtained from LEDGF/p75-deficient cells (si1340/1428 cells) and transfected with plasmid expressing SSRP1-Myc and HIV-1 IN-Myc, and either FLAG-LEDGF/p75 (*lane 1*) or an empty plasmid (*lane 2*) (**c**). Then, immunoprecipitated proteins were evaluated for the presence of the expressed proteins by immunoblotting with tag-specific antibodies. (**) represents a longer exposure of (*). Detection of light chain Igs were used as loading control. The experiment was performed twice with identical results

FACT is a histone chaperone heterodimeric complex composed of human homolog of the suppressor of Ty16 (hSpt16) and the structure-specific recognition protein 1 (SSRP1) [17]. This chromatin remodeling complex is tightly associated with the PolII transcription machinery [17–19] and was previously shown to participate in the regulation of HIV-1 LTR-driven transcription [20]. Moreover, a recent study reported that the SSRP1 FACT component interacts with LEDGF/p75 via their HMG and PWWP domains [21] (a summary of the previously identified interactions is shown in Fig. 1a). The close proximity found between SSRP1 and LEDGF/p75 protein in cells strongly supports the enrichment of FACT in the chromatin regions targeted by HIV-1 integration complexes [21]. Consequently, in view of (i) the histone chaperone activity of FACT, (ii) the previously reported links between this complex and HIV-1 replication and (iii) the importance of chromatin remodeling in regulating HIV-1 integration we further analyzed its potential function in modulating the insertion of viral DNA into chromatin.

## Results

### Selection of cellular binding partners of HIV-1 IN•DNA complex

To identify new integration partners, we used a pull-down strategy depicted in Additional file 1: Figure S1. Fractions enriched in IN•U5 viral DNA end nucleocomplexes were generated under previously reported conditions [22] using recombinant IN and viral DNA fragments fused to a biotin in their 5′ end. IN•viral DNA complexes were checked by in vitro concerted integration activity (see Additional file 1: Figure S1). These fractions were then incubated with cellular protein extracts from HIV-1 permissive HeLa P4 cells previously counter-selected against beads coupled to DNA alone to limit the unspecific selection of potential DNA and avidin binders. Cellular partners were then sorted using magnetized streptavidin-coupled beads. The sorted proteins were analyzed on SDS-PAGE and compared to proteins found to be non-specifically associated with beads coupled to the viral DNA fragment without IN. As shown in Additional file 1: Figure S1, the experiments performed using the IN•U5 complex led to an apparent enrichment of cellular factors when compared to conditions using beads coupled to U5 DNA without IN. The cellular proteins selected under each condition were further digested *in gel* by trypsin and peptides analyzed by Liquid chromatography coupled to tandem mass spectrometry (LC–MS/MS). Experiments were conducted in triplicate and a set of about 75 proteins specifically and systematically found to be associated with the IN/DNA complex and not with control DNA alone were selected.

Among the selected proteins, most of the IN cofactors or proteins previously associated with integration were also selected under these conditions including LEDGF/p75, INI1, VBP1, FEN1, BAF, and RAD51 [12, 23–25], thereby validating our approach. In addition to these previously identified factors, several other proteins were also selected. These proteins were found to be mainly associated with nuclear import, DNA repair, protein degradation, and chromatin maintenance pathways (see Table 1 reporting the main selected proteins and complementary list Additional file 2: Table S2). In the present work, only the selected proteins associated with chromatin or transcription were considered in view of the impact of the chromatin structure on integration. Interestingly, most of these proteins were also found to be associated with the PolII transcription machinery. Due to (i) the possible modulation of HIV-1 integration by chromatin remodeling, (ii) the recently reported interaction between FACT and LEDGF/p75 IN cofactor and (iii) the enrichment of FACT in transcribed region of the chromatin [26], we investigated the potential role of this complex on HIV-1 integration.

**Table 1:** Selection of cellular interact ants of the IN•viral DNA complex

Protein name	Protein family	Protein complex	Accession number	Number of peptide
LEDGF/p75	Transcription	PolII	O75475	4
PFD3 (VBP1)	Protein chaperone	Prefoldin PFD	F5H2A7	12
SMARCB1 (INI1)	Transcription	SWI/SNF	B5MC5	3
BAF	DNA binding/chromatin	–	O75531	2
FEN1	DNA repair	–	P39748	15
RAD51	DNA repair	–	Q06609	7
SSRP1	Histone chaperone	FACT	E9PPZ7	10
SPT16	Histone chaperone	FACT	Q9Y5B9	31
IWS1	Transcription	PolII	E7EX51	2
SMARCC1 (BAF155)	Transcription	SWI/SNF	Q92922	2
SMARCD1 (BAF60)	Transcription	SWI/SNF	Q96GM5	2

Cellular extracts from HeLa P4 cells were incubated with streptavidin beads coupled to fraction enriched in active IN•viral DNA complexes as shown in SI. The elution of the interacting proteins was loaded on 1256 SDS-PAGE gel stained with silver nitrate and the bands were excised from gel and submitted to electroelution. The selected proteins were identified by MS–MS. A list of proteins found only associated to IN•viral DNA complexes but not to control DNA alone and linked to chromatin or transcription is provided here. The number of peptides identified per protein is also reported. Selected proteins previously reported as playing a role in retroviral integration are underlined

### FACT complex forms a tri-partite complex with HIV-1 IN and LEDGF/p75 both in vitro and in cells

We first checked by in vitro co-immunoprecipitation whether the selection of FACT was due to a direct or indirect interaction between the complex and the retroviral IN. As reported in Fig. 1b (see representative experiments in Additional file 3: Figure S3), no direct interaction was observed between the purified recombinant FACT complex and IN. Since SSRP1 has been shown to bind LEDGF/75, we tested whether FACT could interact with the recombinant purified IN•LEDGF/p75 complex. Unlike IN alone, a reproducible association between the IN•LEDGF complex and FACT was detected, indicating that the the three proteins can form a complex altogether in vitro. This finding is supported by the interaction between FACT and LEDGF/p75-GST detected in GST-pull down experiments (Fig. 1b and Additional file 3: Figure S3). To determine whether the IN/LEDGF/FACT interaction occurs via the LEDGF/SSRP1 interaction we used a 326–471 amino-acid LEDGF/p75-GST construct lacking the PWWP SSRP1 interacting domain (as determined previously, [21]) but carrying the integrase binding domain (IBD). We first confirmed that the IBD interacts with HIV-1 IN but not FACT by GST pull down (Additional file 3: Figure S3). As shown in Fig. 1b, no interaction was detected between IN/IBD/FACT by co-immunoprecipitation suggesting that the formation of the IN/LEDGF/FACT complex requires the previously reported physical association between FACT and LEDGF/p75 mediated by SSRP1 and PWWP domains [21].

To define whether LEDGF/p75 also forms a complex with HIV-1 integrase and components of the FACT complex in cells, lysate from LEDGF/p75-deficient HEK293T cells transiently transfected with plasmids expressing Myc-tagged SSRP1 and HIV-1 IN, and either FLAG-tagged LEDGF/p75 or an empty plasmid were subjected to immunoprecipitation with anti-FLAG antibodies. The presence of these proteins in the immunoprecipitated samples was evaluated by immunoblot with tag-specific antibodies. In support to our in vitro findings, data in Fig. 1c indicate that SSRP1 and IN strongly associated with LEDGF/p75 in cells. The FACT subunit SSRP1 was sufficient for the formation of this complex, indicating its Spt16-independence. Similarly, SSRP1 was also reported to be sufficient to associate the FACT complex to LEDGF/p75 [21]. Low levels of HIV IN were observed in the control cells lacking LEDGF/p75 (lane 2, Fig. 1c) as expected considering the inhibitory effect of LEDGF/p75 on the proteasome-mediated HIV-1 IN degradation previously reported [27].

Based on these identified interactions we next investigated the effect of FACT on the integration process both in vitro and in cells.

### FACT-dependent nucleosome disassembly promotes HIV-1 integration into chromatin in vitro

**Fig. 2:**
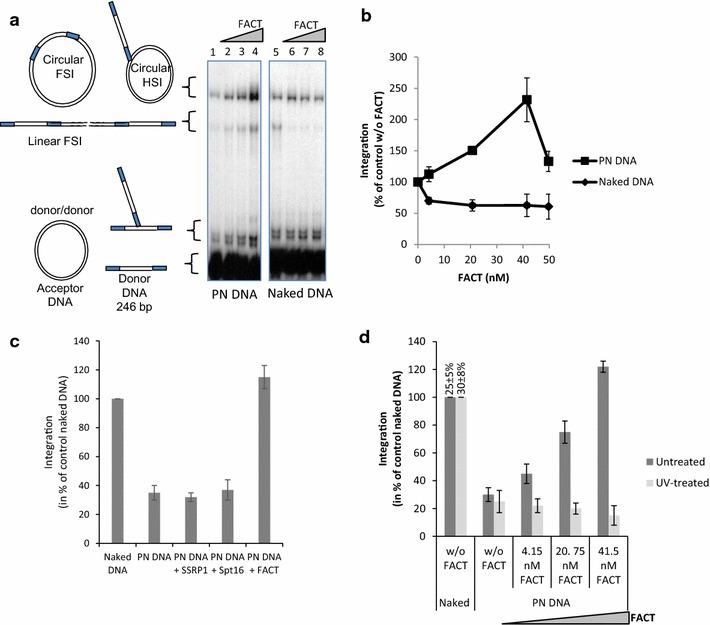
Effect of FACT complex on in vitro HIV-1 integration. A concerted integration assay was performed using 200 nM of HIV-1 IN, 10 ng of donor DNA, 50 ng of p5S naked or chromatinized plasmid DNA (PN DNA), and increasing concentrations of FACT complex. The reaction products were loaded onto 1% agarose gels, and a representative set of experiments performed with the wild-type integrase is reported in (**a**). The positions and structures of the donor substrate and the different half-site (HSI), full-site (FSI) and donor/donor integration (d/d) products are shown. Quantification of the total integration products (FSI, HSI and donor/donor) was performed via gel detection and is reported in (**b**) as the percentage relative to activity without FACT. Effect of FACT complex or SSRP1 or Spt16 proteins [optimal FACT concentration as determined in (**b**)] on integration into PN DNA was analyzed and integration efficiency was reported in (**c**). Effect of FACT nucleosome remodeling activity on integration catalyzed by HIV-1 IN on chromatinized substrates was analyzed by comparing chromatinized p5S vector (histone/DNA ratio = 1) treated with UV or untreated as reported in “Methods” section. The treated or untreated substrates were then used in concerted integration assays without FACT or with increasing concentrations of the complex. Quantification of the total integration products detected on gel (see representative experiment in Additional file 4: Figure S4) (FSI, HSI and donor/donor) was performed via gel detection and is reported in (**d**) as the percentage relative to activity detected on naked DNA (pre-normalized data obtained in the control experiments using naked DNA and without FACT are also reported as the percentage of integrated substrate). All values are shown as the mean ± standard deviation (*error bars*) of at least three independent sets of experiments

While HIV-1 IN integrates efficiently in mononucleosomes [4, 5] the concerted full site integration was found to be inhibited by nucleosome compaction in vitro [8, 14]. We, thus, tested the effect of FACT-dependent nucleosome disassembly on in vitro integration performed in reconstituted chromatin templates. Recombinant purified FACT complex was added in a typical concerted integration assay using polynucleosomal substrates (PN) and HIV-1 IN. As reported in Fig. 2a, b, while nucleosomes assembled as dense and stable chromatin were found to be refractory to HIV-1 integration (c.f. lanes 1 and 5), the addition of FACT restored integration in the chromatinized template (lanes 2–4) reaching a higher integration efficiency than found in naked DNA (c.f. lanes 4 and 5). Notably, optimal integration stimulation was obtained with a 1–2 nucleosome/FACT ratio which correlates well with the optimal ratio previously reported for the action of FACT on chromatin dissociation during transcription stimulation [17]. Increasing the FACT concentration led to a decrease in integration efficiency confirming that an optimal amount of the complex is required for the integration promotion (see Fig. 2b). Importantly, in contrast to what is observed on the chromatinized templates, the addition of FACT on the concerted integration assay using naked DNA induced a minor inhibition that may be due to competition with DNA (indeed, pre-incubation between FACT and naked acceptor DNA increased this inhibition effect, data not shown). Furthermore, the restoration effect was not observed when using SSRP1 or SPT16 alone (Fig. 2c). This result indicates that the restoration of integration observed on chromatinized DNA was dependent on the presence of nucleosomes and required a fully assembled active FACT complex.

To better understand the molecular mechanism of the integration restoration induced by FACT we investigated the structure of the chromatin after template treatment. To this purpose a typical Formaldehyde-Assisted Isolation of Regulatory Elements (FAIRE) approach was used as previously reported [28] and described in Additional file 4: Figure S4. Data show that FACT increases DNA accessibility in treated chromatinized templates suggesting that this chromatin remodeling activity could be responsible for the integration restoration. To confirm this hypothesis, we prevented the FACT-dependent histone/DNA dissociation by inducing protein-DNA crosslinks with UV in the acceptor DNA before integration assays. FAIRE analysis of templates pre-treated by UV and then submitted to FACT activity confirmed that UV crosslinking strongly inhibits FACT-dependent nucleosome remodeling (Additional file 4: Figure S4). Integration assays performed using these UV-treated templates showed that the crosslinking also abolished the capability of FACT to restore integration in PN (Fig. 2d and typical experiment in Additional file 4: Figure S4). Taken together, these data showed a strong correlation between the FACT-mediated restoration of HIV-1 integration and its nucleosome dissociation activity.

### LEDGF/p75 potentiates the FACT-mediated restoration of HIV-1 integration

**Fig. 3:**
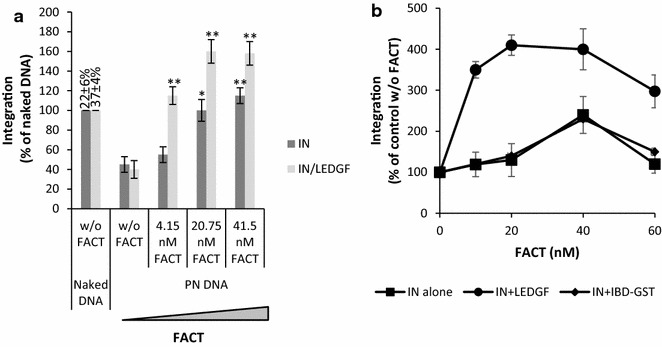
Effect of LEDGF/p75 on FACT-mediated stimulation of in vitro HIV-1 integration. Concerted integration assays were performed as done in Fig. 2 but using IN•LEDGF complex, naked or chromatinized acceptor DNA and increasing concentrations of FACT (**a**). Results are reported as the percentage relative to activity detected on naked DNA without FACT and pre-normalized data obtained in the control experiments using naked DNA and without FACT are reported as the percentage of integrated substrate. Results obtained with full length LEDGF/p75 or the LEDGF 326–471 IBD-GST domains are reported in (**b**). All values are shown as the mean ± standard deviation (*error bars*) of at least three independent sets of experiments. The p-values were calculated by Student’s t-test and are shown as *p < 0.05 and **p < 0.005 to represent the probability of obtaining significant differences compared with control experiments performed with PN DNA without FACT

We next addressed whether the presence of LEDGF/p75 could modulate the effect of FACT on integration. As reported in Fig. 3a, the purified IN•LEDGF/p75 complex was also inhibited by the chromatinization of the acceptor template. Addition of FACT also restored integration catalyzed by the IN•LEDGF/p75 complex on PN templates and the observed restoration was more efficient than observed with IN alone. This result suggests that LEDGF/p75 potentiates the effect of FACT on integration. No stimulation of the FACT-mediated remodeling activity was observed in the presence of LEDGF/p75 suggesting that the potentiation effect was not due to the stimulation of FACT activity on chromatin (Additional file 4: Figure S4). To further determine whether the binding of LEDGF/p75 to IN was required for this potentiation we tested the isolated LEDGF/p75 integrase binding domain (IBD) which does not bind to FACT ([21], c.f. Fig. 1). As shown in Fig. 3b, the IBD did not induce the FACT potentiation observed with the LEDGF/p75 full-length protein. Taken together these data strongly support the importance of both LEDGF/p75-FACT and LEDGF/p75-IN interactions in the FACT-mediated restoration of HIV-1 integration.

Interestingly, the integration efficiency in PN at the optimal FACT concentration was higher than that detected on naked DNA. These data suggest that the chromatin structures generated by the action of FACT on polynucleosomes are highly preferential for HIV-1 integration and are even better substrates than naked DNA in vitro. Consequently, we have investigated the impact of these FACT-induced chromatin structures on integration.

### Reconstituted chromatin containing partially dissociated nucleosomes is a favored substrate for HIV-1 integration in vitro

**Fig. 4:**
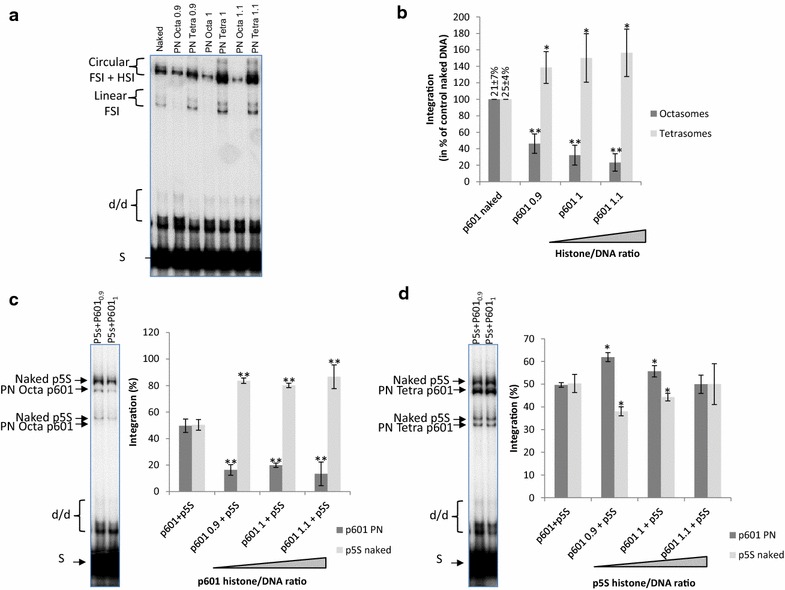
Integration catalyzed by HIV-1 integrase onto dissociated nucleosomes in vitro. A concerted integration assay was performed using 200 nM of HIV-1 IN, 10 ng of donor DNA, 50 ng of p601 naked or p601 plasmid chromatinized either using native histone octamers (PN Oct) or H3/H4 tetramers (PN Tet) with increasing histone/DNA ratio as reported. Typical experiment is shown in (**a**) and quantification of the integration products is reported in (**b**). Results are reported as the percentage relative to activity detected on naked DNA and pre-normalized data obtained in the control experiments using naked DNA and without FACT are reported as the percentage of integrated substrate. Selectivity assays were performed under similar conditions except that a mixture of naked p5S and either p601 assembled with native octamers was used (**c**) or H3/H4 tetramers (**d**). Quantification of the integration products detected in each vector is reported as percentage of the total integration. All values are shown as the mean ± standard deviation (*error bars*) of at least three independent sets of experiments. The p-values were calculated by Student’s t-test and are shown as *p < 0.05 and **p < 0.005 to represent the probability of obtaining significant differences compared with untreated conditions

The remodeling activity of the FACT complex has been extensively studied and some studies have shown the FACT-dependent eviction of H2A/H2B dimers from the native nucleosome [18]. We, thus, wondered whether the FACT-dependent activation of integration in reconstituted chromatin was due to a local increase in targeted sites mediated by the eviction of H2A/H2B dimers by the remodeling complex. Chromatin assembled with H3/H4 tetramers, instead of H2A/H2B/H3/H4 octamers, forms tetrasomal templates (PN Tetra) that share structures similar to the FACT remodeled products [29]. Thus, these PN Tetra templates allow to mimic the chromatin structures enriched in PolII transcribed regions, and to test their capacity as integration substrates. Consequently, we tested PN Tetra in HIV-1 integration and compared the integration efficiency with naked DNA or chromatin assembled with native histone octamers (PN Octa). The pBSK-601-Zeo vector (p601) containing 601 Widom repetitions for highly stable assembly of nucleosomes was used to generate these templates. As reported in Fig. 4a, polynucleosomes assembled with H3/H4 tetramers were always found to be better substrates for integration than chromatin fully assembled with histone octamers. Interestingly, these tetrasomal nucleosomes were also found to be better substrates than the corresponding naked DNA regardless of the ratios used (see quantification in Fig. 4b).

To confirm the preference of HIV-1 IN for partially dissociated nucleosomes, we performed an in vitro selectivity assay as set up previously [15] using a mixture of naked and chromatinized Octa or Tetra templates. Integration was clearly preferred in the naked DNA when mixed with PN Octa templates (Fig. 4c). In contrast, when a mixture of naked DNA and PN Tetra substrates was used (Fig. 4d), integration was preferred in the PN Tetra templates, thereby confirming the preference of HIV-1 for these structures over naked DNA or native nucleosome templates.

**Fig. 5:**
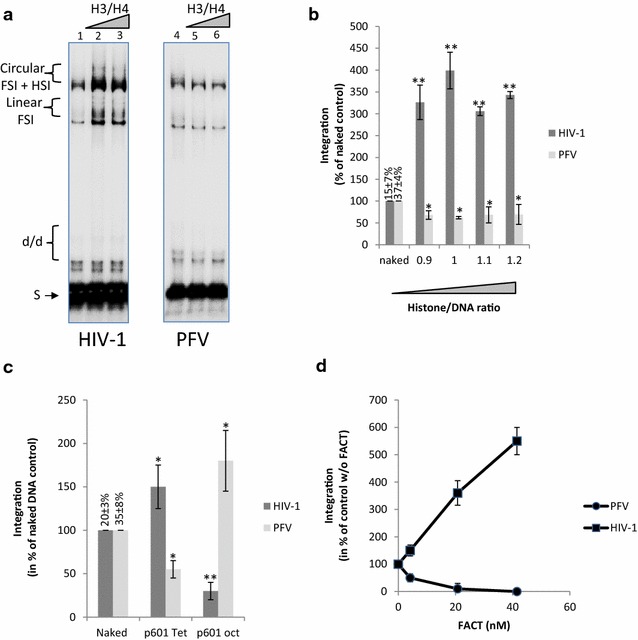
Effect of nucleosome dissociation on integration catalyzed by HIV-1 and PFV integrases in vitro. Concerted integration assays were performed using either HIV-1 or PFV INs under similar conditions and using p601 vectors naked (lanes 1 and 4) or chromatinized with native histone octamers or H3/H4 tetramers with increasing histone/DNA ratios (lanes 2 and 5, 1.1 ratio; lanes 3 and 6, 1.2 ratio). Typical experiment is shown in (**a**) and quantification of the integration products is reported in (**b**). Comparison of the integration catalyzed by HIV-1 or PFV IN on naked and p601 vectors assembled with histone octamers or tetramers is shown in (**c**). Results are reported as the percentage relative to activity detected on naked DNA without FACT and pre-normalized data obtained in the control experiments using naked DNA are reported as the percentage of integrated substrate. Comparison of the effect of FACT on HIV-1 and PFV integration catalyzed on p601 chromatinized with histone octamers is shown in (**d**). All values are shown as the mean ± standard deviation (*error bars*) of at least three independent sets of experiments. The p-values were calculated by Student’s t-test and are shown as *p < 0.05 and **p < 0.005 to represent the probability of obtaining significant differences compared with untreated conditions

Since PFV intasome was shown to require the binding to H2A/H2B histones for efficient docking to the nucleosome [7], we next compared HIV-1 and PFV IN on the evicted and remodeled templates. As reported in Fig. 5a and b, while PN Tetra are good substrates for HIV-1 integration, they are rather ineffective with PFV. In addition, while FACT stimulates HIV-1 integration into PN Octa templates, its remodeling activity clearly inhibits PFV integration in the same templates (Fig. 5c). This inhibition confirmed the poor integration efficiency found for PFV IN on partially dissociated chromatin and the requirement of this retroviral system for native nucleosomes, as suggested by the structure of the PFV intasome/nucleosome complex [7].

Taken together, these results strongly suggest that in vitro efficient HIV-1 integration into chromatin requires the presence of partially dissociated nucleosomes as generated by FACT remodeling. We next investigated the effect of FACT in the context of infected cells.

### Cells with enhanced FACT-mediated chromatin remodeling activity are more permissive to HIV-1 infection and integration

The FACT complex has a dual activity on nucleosomes during polII transcription since it induces a partial dissociation of the histones, thereby allowing the polymerase to travel across nucleosomes, and participates in their re-association after elongation [17, 18, 30, 31]. We thus investigated how these activities exerted in preferred integration regions could influence viral replication.

**Fig. 6:**
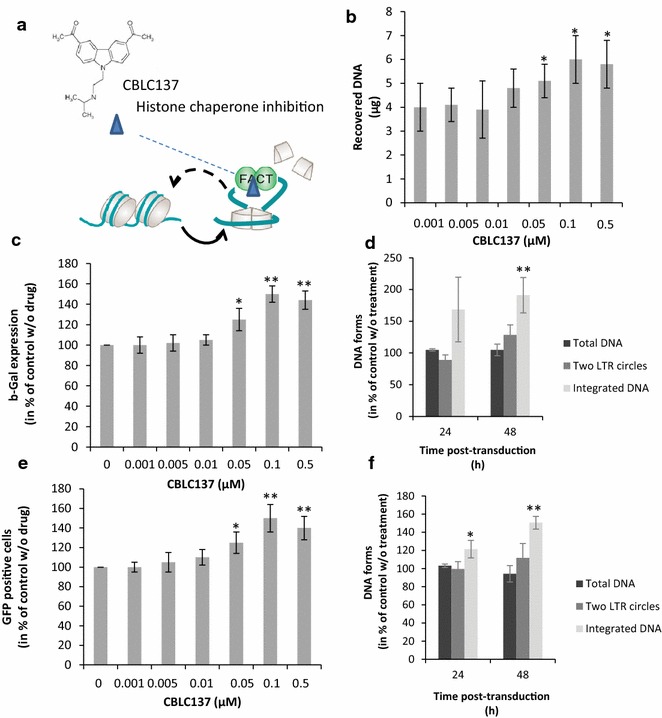
Effect of FACT-mediated chromatin remodeling chemical promotion on early steps of HIV-1 LAI virus and derived lentiviral vectors. HeLa P4 and HEK293T cells were treated with CBLC137 curaxin 6 h before cell infection with LAI wild type virus or transduction with lentiviral vectors. The structure of the CBLC137 drug is shown in (**a**) as well as its effect on FACT. CDLC137 binds to nucleosomal DNA and forms structures with high FACT affinity leading to the trapping of the complex into dissociated nucleosomes, preventing its histone chaperone activity and enhancing the histone dissociation [32, 33]. This induces a global increase in chromatin accessibility that was evaluated by FAIRE (data obtained in HEK293T cells are reported in **b**). The effect on LAI virus replication was evaluated by quantifying the LTR-dependent β-galactodisase expression (**c**) and viral DNA populations at 0–48 h post-transduction (see 24 and 48 h time points in **d** and full time course analysis in Additional file 6: Figure S6). Early steps of lentiviral vector replication were evaluated by quantifying GFP-positive HEK293T cells (**e**) and viral DNA populations at 0–48 h post-transduction (see 24 and 48 h time points in **f** and full time course analysis in Additional file 6: Figure S6). Quantification of the viral DNA populations were done after optimal 0.1 µM curaxins treatment. All values are shown as the mean ± standard deviation (*error bars*) of at least three independent sets of experiments. The p-values were calculated by Student’s t-test and are shown as *p < 0.05 and **p < 0.005 to represent the probability of obtaining significant differences compared with untreated conditions

FACT-dependent chromatin remodeling can be enhanced by both genetic and pharmacologic approaches. Curaxins drugs, as CBLC137, have been previously shown to causes partial unwrapping of DNA from the nucleosome core leading to the dissociation of the H2A/H2B dimer and the exposure of the H3/H4 tetramer docking surface for FACT binding [32]. Binding of FACT to this pre-dissociated nucleosomal structure induces its trapping onto the nucleosome, inhibits its chaperone activity and enhances the nucleosome dissociation leading to a global increase in chromatin accessibility (29, and Fig. 6a). Consequently, curaxins treatment was used to study the effect of FACT-mediated chromatin remodeling on the early steps of viral replication. CBLC100 and CBLC137 compounds previously described to induce the FACT nucleosomal trapping [32, 33] were used. Both molecules were first assayed on in vitro integration catalyzed by recombinant HIV-1 IN in order to exclude direct effects on the protein. As shown in Additional file 5: Figure S5, while CBLC137 showed no or little effect on IN, CBLC100 inhibited directly the retroviral enzyme in the 0.1–1 µM range. Measurement of the cytotoxicity of the drugs in all our cell models showed a modest effect at concentrations below 200 nM, the CBLC137 compounds being the least effective (Additional file 5: Figure S5). For these reasons we selected the CBLC137 molecule for further analysis of its effect on the early steps of viral replication. We first analyzed the effect of curaxin treatment on the structure of cellular chromatin. To this end, the accessibility of genomic DNA was determined after curaxin treatment using the FAIRE approach as previously reported [28]. As shown in Fig. 6b, treatment of HEK-293T cells with CBLC137 led to a global increase in free chromatin DNA, as reported before [33], confirming the inhibition of the FACT histone chaperone activity probably due to its trapping onto dissociated nucleosomes as previously observed [32, 33]. Similar results were obtained in different cell lines as K562 and HeLa P4. We next analyzed the impact of curaxin treatment on the retroviral cycle. We first evaluated the infectivity of LAI wild type virus in HeLaP4 cells treated with CBLC137. As shown in Fig. 6c a stimulation of LTR-dependent β-galactosidase expression was observed in HeLa P4 cells treated with CBLC137 and infected with the virus. Quantification of viral DNA population confirmed that integration was stimulated in this system (Fig. 6d and Additional file 6: Figure S6). In order to avoid biases linked to the previously reported regulation of the LTR-driven transcription by FACT [20, 21] and analyze specifically the effect of the complex on the integration step, we next used a single round 293T cells model transduced by a pRRLsin-PGK-eGFP-WPRE VSV-G pseudotyped lentiviral vector. In this system the eGFP expression is independent from the viral LTR and depends on PGK promoter that is not sensitive to FACT. Quantification of the number of eGFP-positive cells by flow cytometry showed a significant and reproducible 1.4 to 1.8-fold increase for cells treated by the drug (Fig. 6e). Quantification of the viral DNA populations 0–48 h post-transduction indicated that the amount of DNA integrated was increased while no significant change was detected in either the total DNA or the two LTR circles (Fig. 6f, and Additional file 6: Figure S6).

**Fig. 7:**
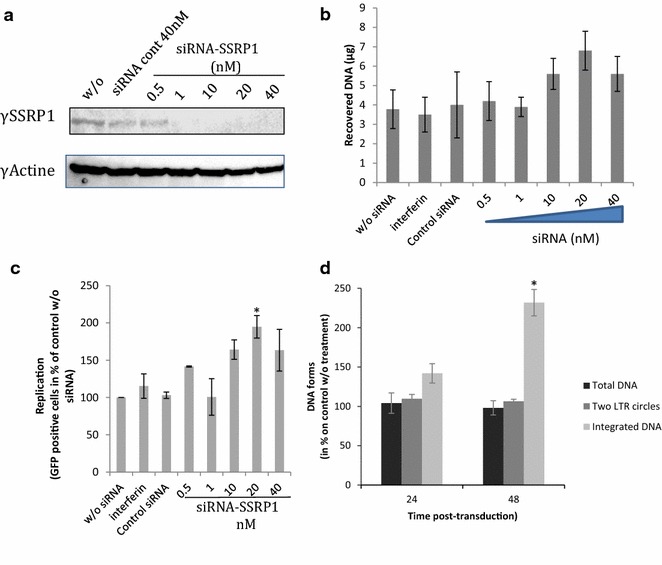
Effect of FACT knock down on early steps of HIV-1 derived lentiviral vectors in HEK293T cells. SSRP1 encoding gene knock down was conducted essentially as previously described [31] by performing two successive siRNA transfections (see “Methods” section). The amount of SSRP1 protein monitored by western blotting is reported in (**a**). This method allowed us to achieve more than 80% of apparent SSRP1 extinction. After knock down, FAIRE analyses were performed as indicated in “Methods” section and the results obtained in cells depleted for SSRP1 are reported in (**b**). The early steps of replication of lentiviral vectors carrying the GFP encoding gene were determined by measuring GFP-positive cells with flux cytometry (**c**) and quantification of the viral DNA populations at 0–48 h post-transduction using quantitative PCR (data obtained after a 20 nM siRNA treatment are reported in **d** for the 24 and 48 h time points, see full time course analysis in Additional file 7: Figure S7). Data obtained with the interferin transfector agent alone and control siRNA are also reported in the figure. All values are shown as the mean ± standard deviation (*error bars*) of four independent sets of experiments. The p-values were calculated by Student’s t-test and are shown as *p < 0.05 and **p < 0.005 to represent the probability of obtaining significant differences compared with untreated conditions

Knockdown of the SSRP1 encoding gene by siRNA has also been shown to increase the amount of FACT-remodeled chromatin sites in highly transcribed genes [26]. Consequently, we also performed additional siRNA knockdown of the SSRP1 encoding gene to analyze the importance of active FACT amount in viral infectivity. SSRP1 encoding gene knock down was conducted essentially as previously described [31]. Two successive transfections of siRNA anti-SSRP1 performed as described in “Methods” section allowed us to achieve about 80% of apparent SSRP1 extinction (Fig. 7a). SSRP1 knockdown also induced a global increase in free chromatin DNA (see FAIR analysis in Fig. 7b) accompanied with an increase of viral infectivity (Fig. 7c) and integration efficiency (Fig. 7d and Additional file 7: Figure S7). Interestingly, siRNA suboptimal concentrations (>20 nM) decreased the integration efficiency suggesting, as observed in vitro, that optimal FACT amounts could be required for the promotion of integration step.

The constant correlation found between the FACT-mediated increase in global accessibility to DNA detected in cells either treated by curaxin, or depleted in SSRP1, prior to transduction, and the efficiency of integration strongly suggests that this step is modulated by FACT chromatin remodeling activity.

### Stimulation of FACT-mediated chromatin remodeling promotes HIV-1 integration by a LEDGF dependent mechanism

**Fig. 8:**
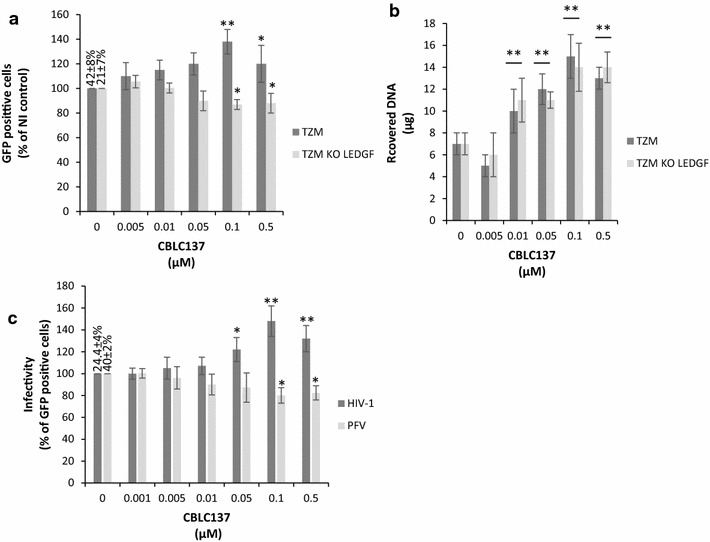
Effect of FACT-mediated chromatin remodeling chemical promotion on early steps of HIV-1 in LEDGF/p75 knock-out context and on PFV infectivity. Similar experiments than described in Fig. 7 were performed in TZM and TZM knock out for LEDGF/p75 (**a**, **b**) or in HEK293T cells transduced either with HIV-1 or PFV vectors (**c**). The effect on the early steps of lentiviral vector replication was evaluated by quantifying GFP-positive cells. All values are shown as the mean ± standard deviation (*error bars*) of at least three independent sets of experiments. The p-values were calculated by Student’s t-test and are shown as *p < 0.05 and **p < 0.005 to represent the probability of obtaining significant differences compared with untreated conditions

Since LEDGF/p75 has been shown to bind FACT and promotes its effect on in vitro integration we have investigated the importance of this factor in the FACT-mediated regulation of viral integration in cells. For this purpose we analyzed the viral infectivity in TZM cells and TZM cells knockout (KO) for LEDGF/p75 [34] and affected for FACT activity. Accumulation of LEDGF knockout and FACT knockdown by siRNA did not allow us to find conditions allowing the analysis of the viral infectivity in cells by this method without biases due to different cellular growth. However, the curaxin-based pharmacological approach allowed us to obtain conditions compatible with this analysis. Parental TZM cells and TZM cells knockout for LEDGF/p75 were thus treated by CBLC137 prior to transduction with a pRRLsin-PGK-eGFP-WPRE VSV-G pseudotyped lentiviral vector. Viral infectivity was then measured. As reported in Fig. 8a, curaxin treatment also induced a stimulation of viral infectivity in TZM cells. In contrast no stimulation was detected in TZM cells LEDGF/p75 KO while CBLC137 treatment induced the expected increase in chromatin accessibility in both cell lines (Fig. 8b). The LEDGF/p75-dependent effect of curaxins on viral infectivity suggests that this factor is required for the integration modulation by FACT in cells. To confirm this we tested the effect of curaxin on the early steps of replication of PFV vector which is not expected to be dependent on LEDGF/p75 and whose integration was found to be inversely regulated by FACT in vitro (see Fig. 5). As reported in Fig. 8c, curaxin treatment did not stimulate PFV infectivity in HEK 293T cells in contrast to HIV-1 vector and even a slight inhibition was detected at high CBLC137 concentrations in agreement with the in vitro data.

## Discussion

Here we report the role of the FACT histone chaperone complex in modulating HIV-1 integration by affecting the structure of chromatin. In cell, FACT controls nucleosome deposition on DNA and has been implicated in many processes involving chromatin, such as transcription, DNA replication, recombination and repair [18, 30, 35]. Interestingly, recent reports show that the FACT complex can bind the LEDGF/p75 HIV-1 integrase cofactor via SSRP1 [21] and could participate in the modulation of viral gene expression [20]. In these studies no effect of FACT on the integration step could be detected. However, the analyses presented by these authors were focused on the viral LTR-dependent gene transcription. In contrast, possible regulation of the retroviral integration mechanisms by FACT has been recently proposed as SSRP1 depletion was shown to inhibit Avian Leukosis Virus (ALV) integration [36]. In this latest work no inhibition of HIV-1 integration by SSRP1 depletion was observed suggesting that FACT-dependent modulation of integration could depend on the retrovirus family. This different regulation of HIV-1 integration by FACT was demonstrated in our work showing a clear stimulation of this replication step when the FACT remodeling activity was stimulated. Interestingly, a stimulation of HIV-1 integration after SSRP1 knockdown was also observed by Winans et al. [36] but was found non-statistically relevant. However, these data were obtained in chicken DT40, which are not naturally permissive to HIV-1 infection, and collected at 24 h post transduction while our data indicate that the increase in integrated DNA amount becomes more relevant a 48 h than 24 h (see Additional file 7: Figure S7).

The possible difference in the retroviral integration regulation mechanism by FACT was confirmed by our data showing that HIV-1 integration regulation by this complex depends on the presence of nucleosomes and chromatin remodeling activity while the activation of integration observed for ALV does not depend on the presence of nucleosomes but on the direct binding of SSRP1 on ALV integrase. These observations contrast with the role of nucleosomes on FACT-dependent activation of HIV-1 integration and the absence of direct interaction between HIV-1 IN and FACT, as presented by our studies. These differences suggest that the mechanism of FACT modulation of integration may depend on the retrovirus and differs between HIV-1 and ALV. In the case of HIV-1, the FACT mediated activation of integration is also modulated by LEDGF/p75 IN partner. We show here that the IN•viral complex can pull the FACT complex down from a cellular protein extract in a LEDGF/p75-dependent manner and the IN•LEDGF complex is able to bind the recombinant FACT in contrast to IN alone. This indicates that IN/LEDGF/FACT interactions are not mutually exclusive and can thus occur simultaneously in the physiological integration complex. These data also suggest that the FACT complex can be loaded at the integration sites where integration and transcription machinery can meet. The stimulation of FACT effect on integration by LEDGF/p75 confirmed the modulation of FACT-dependent regulation of the viral DNA insertion by a mechanism that may involve the LEDGF/SSRP1 interaction. This is additionally supported by the lack of effect of the isolated IBD domain of LEDGF lacking the SSRP1 interaction domain.

We previously reported that while HIV-1 integration into isolated nucleosomes is efficient, their compaction in dense chromatin restricts the reaction and their remodeling can overcome this restriction [8, 14]. Since FACT is associated with the PolII-transcribed regions of chromatin and is closely linked to LEDGF/p75, we made the hypothesis that its remodeling activity could regulate the access of the HIV-1 Intasome to nucleosomes. The observed stimulation of HIV-1 integration in cells where FACT-mediated chromatin remodeling was promoted by curaxins treatment confirmed our hypothesis. As previously shown [32, 33], FACT trapping on chromatin induced by curaxins both reduces the re-association of nucleosomes after PolII machinery elongation and enhances the nucleosomes dissociation leading to an increase in the global amount of open chromatin, especially in the transcribed regions targeted by HIV-1 intasomes. Given the requirement of open chromatin structure for efficient HIV-1 integration, we propose that the activated integration observed in cells inhibited or knockdown for FACT, results from an increased access of nucleosomes for the HIV-1 intasome as supported by our in vitro analysis. Indeed, in vitro integration assays performed in PN templates showed that FACT remodeling activity allowed efficient HIV-1 integration into dense chromatin that was initially refractory to viral DNA insertion. Importantly, this FACT-mediated activation of integration was found to be nucleosome-dependent since no stimulation was detected on naked DNA, in contrast to what was recently observed for ALV [36]. Our data indicate that FACT generates chromatin structures that are highly preferential for in vitro integration. This restoration process was stimulated by the presence of LEDGF/p75, suggesting that its association with FACT may promote the restoration of integration. Based on the direct interaction between FACT and LEDGF/p75, we can speculate that this association may increase the local FACT concentration around integration sites leading to a coupling between nucleosome remodeling and viral DNA insertion.

Additionally, the use of tetrasomes that lack H2A/H2B dimers and mimic the FACT-generated nucleosome structures along transcribed genes allowed us to demonstrate that chromatin containing these partially dissociated forms is a preferential substrate for in vitro HIV-1 integration, and is even better than naked DNA. Interestingly, PFV integrates less efficiently in the dissociated nucleosomes than in the fully structured ones. These data correlate well with the requirement of direct interactions between the PFV intasome and H2A/H2B dimers for optimal integration [7] as well as with the preference of this virus to integrate more often in regions of high nucleosome density [8]. This validates the specificity of our model and highlights the different behavior of HIV-1 and PFV intasomes, as previously reported [8]. These models very likely require distinct constraints for integrating into chromatin and could thus have a distinct active structure at the surface of the targeted nucleosome. This is supported by the various intasome structures reported recently in the literature showing distinct 3D features [4, 35–39]. These distinct preferences for chromatin structures observed for the different retrovirus could also explain their different sensitivity to FACT chromatin remodeling as detected for HIV-1, PFV and ALV. Our biochemical data also suggest that an optimal FACT amount is required for reaching an equilibrium between the nucleosome dissociation and their re-association suitable for integration. This would explain why the FACT depletion in cell allows to reach a local concentration of the complex suitable for the nucleosome dissociation and the integration facilitation. This is fully supported by our FAIR results indicating that under the FACT knockdown or inhibition conditions the chromatin opening is favored.

**Fig. 9:**
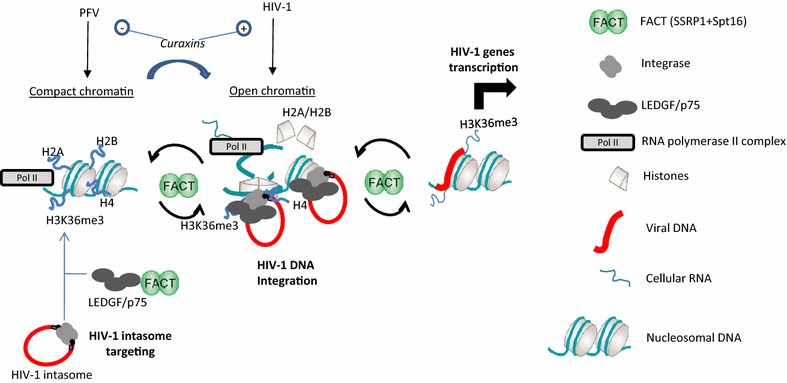
Model for FACT action on HIV-1 integration steps. HIV-1 intasomes are targeted to the PolII-transcribed region of the chromatin thanks to the association of IN with LEDGF/p75 and binding to H3K36me3. In these regions the FACT complex and LEDGF/p75 are both enriched owing to the LEDGF/p75 and SSRP1 interaction. Chromatin remodeling mediated by FACT occurring in the vicinity of the targeted region may generate partially dissociated nucleosomes leading to chromatin structures that are preferential substrates for both RNA transcription and HIV-1 integration. FACT action on chromatin at the vicinity of the integration sites may lead to increased accessibility to nucleosomal DNA as well as histone tails. Integration could then occur efficiently on partially dissociated nucleosome or accessible native nucleosome. Further FACT-mediated regulation of the viral genes transcription can then occur. The expected effect of curaxin treatment on HIV-1 and PFV integration is reported on the basis of the drugs effect on FACT chromatin remodeling activity and on the retroviruses preferences for open or compact chromatin structure

Extensive data from the literature, in addition to the results obtained here for HIV-1 integration, suggest that HIV-1 intasomes are targeted to the PolII-transcribed region of chromatin, mostly thanks to LEDGF/p75 and H3K36me3 recognition, where FACT and LEDGF/p75 are both enriched due to the interaction between LEDGF/p75 and SSRP1. This is strongly supported by the enrichment of FACT in the transcription region of chromatin [26] and the enhancement of its recruitment by H3K36 trimethylation [40]. Collectively, these events should lead to an increase in FACT concentration in the vicinity of HIV-1 integration sites as induced in our in vitro integration assays. This was supported by the loss of integration stimulation by curaxin treatment in TZM cells KO for LEDGF/p75. Chromatin remodeling mediated by FACT and occurring in these HIV-1-targeted regions may generate partially dissociated nucleosomes, leading to chromatin structures that are preferential substrates for both RNA transcription and HIV-1 integration. This coupling between FACT remodeling and integration would thus allow efficient integration onto nucleosomes as detected in infected cells. Inhibition of FACT chaperone activity by curaxins or the extinction of SSRP1 expression would therefore be expected to induce an increase in open chromatin in the PolII regions and to stimulate integration, as observed in our experiments. After these effects on integration FACT may then exert its regulation function on viral gene transcription as previously observed [20, 21]. Based on the difference in their preference for distinct chromatin structure we propose that FACT deregulation could induce different and inverted effect depending on the retrovirus as observed by us with HIV-1 and PFV (see Fig. 8c) and by other authors using ALV model [36]. These putative functions of FACT on retroviral integration is recapitulated in Fig. 9.

The molecular mechanism allowing integration onto partially dissociated nucleosomes remains to be fully unraveled. Indeed, the action of FACT on chromatin could increase accessibility to both DNA and to histones. These two parameters could influence HIV-1 integration on nucleosomes, which may require additional protein/protein interactions as supported by the physical contacts between the PFV intasome and protein histones (H2A/H2B) reported before [7]. We can speculate that such interaction between HIV-1 IN and other histones could also occur and would require the local partial dissociation of the nucleosomes induced by FACT to be efficient. Interestingly, in compact chromatin several histone tails such as histone H4 are engaged in interaction with neighboring nucleosomes and are therefore less or maybe not accessible for association with incoming intasomes [41–43]. Local FACT-meditated remodeling of the chromatin could thus allow or promote both protein/protein and protein/DNA interactions within the targeted nucleosomes. This process would be one mechanism allowing the efficient HIV-1 integration into PolII regions in addition to LEDFG/p75 and CPSF6 targeting, histone modifications, and intrinsic chromatin dynamics found in these loci.

## Conclusions

In addition to reporting a new cellular cofactor of HIV-1 integration, this study also demonstrates a potential link between the retroviral integration machinery and the PolII complex. This suggests that HIV-1 integration/transcription are closely coupled, as indicated by the IN/LEDGF/FACT interaction. This would open the way for a new understanding of these viral steps and could lead to new antiviral strategies targeting both integration and transcription.

## Methods

### Proteins, DNA substrates and chemicals

HIV-1 IN, PFV IN, LEDGF, IN•LEDGF/p75 complex and GST-fused proteins were purified following previously published protocols [8, 44–46]. Polynucleosome assembly was performed as previously reported using either recombinant H3, H4, H2A and H2B octamer or recombinant H3, H4 tetramers (New England Biolabs) by gradient salt dialysis on p5S vector described before [8, 14] or pBSK-Zeo-601 plasmid containing a succession of Widom-601 sequences. Nucleosome assembly was checked by DNase I protection, typical restriction enzyme assay (REA) and mono- and di-nucleosome gel shift in 0.8% native agarose gel, as done before [8]. FACT complex was purified as reported before [17, 18] and its activity was checked by chromatin remodeling assay as reported in Additional file 4: Figure S4. Polyclonal anti-HIV-1 IN antibodies were purchased from Bioproducts MD (Middletown, MD, USA). Polyclonal anti-SSRP1 were purchased from Abcam (ab21584) and anti-Spt16 were purchased from Santa-Cruz (sc-28734). Monoclonal anti-LEDGF/p75 were purchased from Bethyl (848A). CBLC100 and CBLC137 FACT curaxins inhibitor were a kind gift from Dr Gurova K.V. [33].

### Selection of IN•viral DNA cofactors and in vitro interactions

Biotinylated IN•viral DNA-enriched fractions were generated by incubating recombinant pure IN with short DNA fragment corresponding to the 21 bp final nucleotides of the U5 viral ends biotin labeled in 5′ under optimized conditions allowing the formation of highly active IN•DNA complex, as done previously [22]. The generated complexes were checked by concerted integration (see Additional file 1: Figure S1) and then incubated with cellular protein extracts from HeLa P4 cells obtained by cell sonication sorted after counter-selection on beads containing only DNA. The cellular interactants were selected after 1 h of incubation at 37 °C with magnetized streptavidin beads coupled to the IN•DNA complexes in an interaction buffer (50 mM HEPES, pH 7.5; 1 µg/ml BSA; 1 mM DTT; 0.1% Tween 20; 10% glycerol; and 100 mM NaCl). After magnetization and washing with the buffer, the interacting proteins were eluted by adding Laemmli protein loading buffer and heating at 95 °C. The eluted proteins were loaded on 12% SDS-PAGE then stained with Silver Nitrate (ProteoSilver Silver stain kit from Promega). The bands corresponding to the selected proteins were digested as described by Allmann et al. [47].

### Mass spectrometry analysis

Online nanoLC-MS/MS analyses were performed using an Ultimate 3000 system (Dionex, Amsterdam, The Netherlands) coupled to a nanospray LTQ Orbitrap XL mass spectrometer (Thermo Fisher Scientific, Bremen, Germany). Ten microliters of each peptide extract were loaded on a 300 µm ID × 5 mm PepMap C_18_ precolumn (LC Packings, Dionex, USA) at a flow rate of 20 µl/min. After 5 min desalting, peptides were online separated on a 75 µm ID × 15 cm C_18_PepMap™ column (LC packings, Dionex, USA) with a 2–40% linear gradient of solvent B (0.1% formic acid in 80% ACN) in 108 min. The separation flow rate was set at 200 nl/min. The mass spectrometer operated in positive ion mode at a 1.8 kV needle voltage and a 42 V capillary voltage. Data were acquired in a data-dependent mode alternating an FTMS scan survey over the range m/z 300–1700 with the resolution set to a value of 60,000 at m/z 400 and six ion trap MS/MS scans with Collision Induced Dissociation (CID) as activation mode. MS/MS spectra were acquired using a 3 m/z unit ion isolation window and normalized collision energy of 35. Mono-charged ions and unassigned charge-state ions were rejected from fragmentation. Dynamic exclusion duration was set to 30 s.

### Database search and results processing

Mascot and Sequest algorithms through Proteome Discoverer 1.3 Software (Thermo Fisher Scientific Inc.) were used for protein identification in batch mode by searching against the UniProt *Homo sapiens* database (65,304 entries, Reference Proteome Set, Release 2012_03). Two missed enzyme cleavages were allowed. Mass tolerances in MS and MS/MS were set to 10 ppm and 0.6 Da. Oxidation of methionine was searched as dynamic modifications. Carbamidomethylation on cysteine was searched as fixed modification. Peptide validation was performed using Percolator algorithm [48] and only “high confidence” peptides were retained corresponding to a 1% False Positive Rate at peptide level. Only proteins with two minimum and distinct peptides were considered in the results.

### In vitro co-precipitation

IN, LEDGF/p75 or IN•LEDGF/p75 (100 nM) were incubated with 80 nM of FACT complex in 10 µl interaction buffer (50 mM HEPES, pH 7.5; 1 µg/ml BSA;1 mM DTT; 0.1% Tween 20; 10% glycerol; and 50–240 mM NaCl) for 20 min on ice and then for 30 min at room temperature. A 15 µl aliquot of either Dynabeads M-280 sheep anti-Rabbit IgG (Invitrogen, ref. 11203D) previously coupled to polyclonal anti-IN antibodies or Dynabeads M-280 sheep anti-Mouse IgG (Invitrogen, ref. 11201D) previously coupled to monoclonal anti-LEDGF/p75, and washed was then added to a total volume of 300 µl interaction buffer and incubated at room temperature for 1 h under rotation. The beads were washed three times with 300 µl interaction buffer and the precipitated products were re-suspended in 8 µl of H_2_O then 2 µl of 5× Laemmli buffer were added, after which they were separated on a 12% gel via SDS-PAGE. Interacting proteins were detected either by Western blot analysis using anti-HIV-1 IN, anti-SSRP1/SPT16 and/or LEDGF antibodies either by direct gel staining using colloidal blue. GST pull down were performed using 4 µg of proteins incubated in 10 µl interaction buffer (50 mM HEPES, pH 7.5; 1 µg/ml BSA; 1 mM DTT; 0.1% Tween 20; 10% glycerol; and 50–240 mM NaCl) for 20 min on ice and then for 30 min at room temperature. 15 µl of glutathione Sepharose 4B beads (GE Healthcare) were washed and diluted in 275 µl of interaction buffer and then incubated for 1 h at room temperature under rotation. The beads were washed three times with 800 µl interaction buffer and the precipitated products were re-suspended in 8 µl of H_2_O then 2 µl of 5× Laemmli buffer were added, after which they were separated on a 12% gel via SDS-PAGE. Interacting proteins were detected either by Western blot using the corresponding antibodies or by direct gel staining using colloidal blue.

### Immunoprecipitation in cells

FLAG-LEDGF/p75, HIV-1 IN-myc and SSRP1-myc expression plasmids [21, 49, 50] were transfected in HEK293T-derived, LEDGF/p75-deficient cells si1340/1428 cells [51] using the calcium-phosphate co-transfection method as described in [21]. Seventy-two hours after transfection cells (~3 × 10^6^) were lysed in 300 µl of CSK I buffer (10 mM Pipes pH 6.8, 100 mM NaCl, 1 mM EDTA, 300 mM sucrose, 1 mM MgCl_2_, 1 mM DTT, 0.5% Triton X-100) containing protease inhibitors (final concentration: leupeptine 2 µg/ml, aprotinin 5 µg/µl, PMSF 1 mM, pepstatin A 1 µg/ml). Cellular lysates were centrifuged at 1000*g* for 6 min at 4 °C and the pellet containing Triton X-100-insoluble proteins and chromatin-bound proteins was re-suspended in 20 µl of CSK II buffer (10 mM Pipes pH 6.8, 100 mM NaCl, 300 mM sucrose, 6 mM MgCl_2_, 1 mM DTT) supplemented with protease inhibitors, 16 units of turbo DNase (Ambion™), 3.4 µl of (NH_4_)_2_SO_4_, and 3.1 µl of 10× turbo DNase reaction buffer. DNase treatment was conducted at 37 °C for 30 min. After incubation, 300 µl of CSK I buffer was added to the DNase treated sample to dilute the (NH_4_)_2_SO_4_ and centrifuged at 22,000*g* for 3 min. Then the supernatant (S2 fraction) was pre-clear twice with goat anti-mouse IgG-coated magnetic beads (magnetized beads, Thermo Scientific, Cat. No. 21354). Then pre-cleared lysates were incubated for 2 h at 4 °C with magnetized beads preloaded with anti-FLAG mAb (Sigma, F3165). Bead-bound proteins were eluted by boiling in Laemmli sample buffer after extensive washing. Then, immunoprecipitated proteins were analyzed by immunoblotting as described in [21]. FLAG-tagged LEDGF/p75 was detected with anti-FLAG mAb (1/500, M2, Sigma) and Myc-tagged SSRP1 and HIV-1 IN were detected with anti-Myc mAb (1/500, clone 9E10, Covance, MMS-150P).

### In vitro integration assays

Typical concerted integration assays were performed as previously reported [8] using 200 nM of IN, 10 ng of donor DNA, and 50 ng of plasmid DNA (reaction solution: 20 mM HEPES pH 7, 15% DMSO, 8% PEG, 10 mM MgCl_2_, 20 µM ZnCl_2_, 100 mM NaCl, 10 mM DTT final concentrations). After the reaction, the resulting integration products were treated with proteinase K 1 mg/ml for 1 h at 55 °C and with phenol/chloroform/isoamyl alcohol (24/25/1, v/v/v). Aqueous phase was then loaded onto a 1% agarose gel. The gel was then dried and submitted to autoradiography. The bands corresponding to free substrate (S), donor/donor (d/d), linear FSI (FSI) and circular HSI + FSI (HSI + FSI) products were quantified by ImageJ software. The circular FSI products were quantified by cloning them into bacteria and determining the numbers of ampicillin-, kanamycin- and tetracycline-resistant clones as percentages of the integration reaction control, which was performed using the wild-type enzyme. UV crosslink was performed by placing 50 ng of chromatinized vectors in 15% DMSO, 8% PEG, 10 mM MgCl_2_, 20 µM ZnCl_2_, 100 mM NaCl, 10 mMDTT final concentration in a 96-well plate. The plate was irradiated under a UV lamp (Bioblock Scientifif) at 254 nM for 10 min at 4 °C before use in concerted integration.

### Cellular procedures

Human Embryonic Kidney 293 (HEK-293T), HeLa and HeLa P4 are typical laboratory cell lines. TZMbl LEDGF knock-out cells [34] were a kind gift of Dr. José Esté from the AIDS Research Institute-Irsicaixa. Lentiviral transductions were performed using pRRLsin-PGK-eGFP-WPRE VSV-G pseudotyped lentiviral vectors as previously described [24]. PFV transductions were performed using single-cycle viruses produced by co-transfection of HEK293T cells (Cell Services, London Research Institute) with pMD9 (GFP reporter PFV vector) and codon-optimized foamy virus GAG, POL and ENV packaging constructs and as previously described [7]. An optimized multiplicity of infection (MOI) of 1 was used, which resulted in 25–35% of the cells containing one copy of proviral DNA as determined before. Fluorescence was quantified 10 days post-transduction by counting 10,000 cells on a FACSCalibur flow cytometer (Becton–Dickinson, San Jose, CA, USA). HIV-1 DNA species were quantified at 8, 24 and 48 h post-transduction as previously described [52]. The total and integrated HIV-1 DNA levels were determined as copy numbers per 10^6^cells. Integrated cDNA and 2-LTR circles were expressed as a percentage of the total viral DNA.

HeLa P4 cells expressing CD4 and CXCR4 receptors, and carrying the stably integrated lacZ gene under the control of the HIV-1 LTR were infected by HIV-1 Lai (1.10^8^ particles/ml, M.O.I = 0.4) as previously reported [24]. Under this system the β-galactosidase activity, whose expression is linked to the expression of the Tat protein, is proportional to HIV-1 integration. In the infection experiments HeLa P4 cells were plated in 48-multiwell plates at 50,000 cells/well using 400 µl of DMEM (Invitrogen, Carlsbad, CA) containing 10% (v/v) fetal calf serum (FCS, Invitrogen) and, 50 µg/ml of gentamycin (Invitrogen). After overnight incubation at 37 °C, medium was replaced with 400 µl of fresh DMEM containing either HIV-1 Lai (1.10^8^ particles/ml, M.O.I = 0.4) produced as described in [53]. After 24 h at 37 °C each well was refilled with 400 µl of a reaction buffer containing 50 mM Tris–HCl pH 8, 100 mM β-mercaptoethanol, 0.05% Triton X-100 and 5 mM of 4-methylumbelliferyl-β-D galactoside (4-MUG) (Sigma, St. Louis, MO). The level of the reaction was measured in a fluorescence microplate reader (Cytofluor II; Applied Biosystems, Foster City, CA) at 360/460 nm Ex/Em after 24 h incubation.

### siRNA transient transfection

293T and HeLa cells (40% confluence in 48 well plates) were transfected with 0.5, 1, 10, 20, and 40 nM of the scramble and SSRP1siRNA (Santa-Cruz sc-37877) using the INTERFERin Polyplus transfection agent. After 24 h (60% confluence), cells were retransfected with 0.5, 1, 10, 20, and 40 nM of the scramble and SSRP1siRNA using the INTERFERin Polyplus transfection agent. Cells were cultured in DMEM with 20% FCS for serum stimulation. The cells were harvested after 96 h for Western blotting. For Western blotting, the cells were lysed with RIPA buffer containing phenylmethylsulphonylfluoride (PMSF) protease inhibitor (0.1 mM) and were subjected to Western blot.

### Formaldehyde-assisted isolation of regulatory elements (FAIRE)

*In cellulo*: Analysis was adapted from previously reported conditions [54]. Four independent cultures (biological replicates) of cells treated or untreated by curaxin or subjected to SSRP1 siRNA were grown in 245 × 245-mm plates to 90% confluence. Formaldehyde was added directly to the plates at room temperature (22–25 °C) to a final concentration of 1% and incubated for 1, 2, 4, or 7 min, respectively. Glycine was added to a final concentration of 125 mM for 5 min at room temperature to quench the formaldehyde. Cells were rinsed with phosphate-buffered saline containing PMSF, and the plate was scraped and rinsed two more times. The cells were spun at 2000 rpm for 4 min and snap-frozen. Cells were resuspended in 1 ml of lysis buffer (2% Triton X-100, 1% SDS, 100 mM NaCl, 10 mM Tris–Cl at pH 8.0, 1 mM EDTA) per 0.4 g of cells and lysed using glass bead disruption for five 1-min sessions with 2-min incubations on ice between sessions. Samples were then sonicated for five sessions of 60 pulses (1 s on/1 s off) using a Branson Sonifier at 15% amplitude. Cellular debris was cleared by spinning at 15,000 rcf for 5 min at 4 °C. DNA was isolated by adding an equal volume of phenol–chloroform (phenol, chloroform, and isoamyl alcohol 25:24:1 saturated with 10 mM Tris at pH 8.0, 1 mM EDTA), vortexing, and spinning at 15,000 rpm for 5 min at 4 °C. The aqueous phase was isolated and stored in a separate tube. An additional 500 μl of TE was added to the organic phase, vortexed, and spun again at 15,000 rpm for 5 min at 4 °C. The aqueous phase was isolated and combined with the first aqueous fraction, and a final phenol–chloroform extraction was performed on the pooled aqueous fractions to ensure that all protein was removed. The DNA was precipitated by addition of sodium acetate to 0.3 M, glycogen to 20 μg/ml, and two-fold the volume of 95% ethanol, and incubated at −20 °C overnight. The precipitate was spun at 15,000 rpm for 10 min at 4 °C, then the pellet was washed with 70% ethanol and dried in a Speed-Vac. The pellet was resuspended in dH_2_O and treated with Rnase A (100 μg/ml) and incubated at 37 °C for 2 h. DNA concentration was evaluated in the pellet and the different supernatant by nanodrop. *In vitro.* 50–100 ng of PN DNA treated or not with FACT were incubated 30 min at 37 °C with 2% formaldehyde. DNA was then sonicated 3 × 30 s and extracted with 50 µl of phenol–chloroform (24/25 v/v) solution followed by a 10-min centrifugation at 13,000*g*. The free DNA concentration in the supernatant was quantified by nanodrop and loaded on 1% agarose gel.

## Additional files

**Additional file 1: Figure S1.** Strategy for selection of cellular interactants of IN•viral DNA complex. Cellular extracts from HeLa P4 cells were first incubated with streptavidin beads coupled to the viral DNA fragments in order to avoid selecting proteins binding solely to the DNA. The elution was then incubated with streptavidin beads coupled to fractions enriched in active IN•viral DNA complexes (**A**). Formation of active IN•DNA complexes was checked in vitro concerted integration (**B**), the data obtained with increasing concentration of INs, 100, 200, 400 nM (lanes 1–3) are reported. The elution of the interacting proteins was loaded on 12% SDS-PAGE gel stained with silver nitrate and the bands were excised and electroeluted. A typical result from a selection performed with the IN•viral DNA and the control DNA alone is reported in (**C**).

**Additional file 2: Table S2.** List of additionally selected proteins. Cellular extracts from HeLa P4 cells were incubated with streptavidin beads coupled to fraction enriched in active IN•viral DNA complexes as shown in Additional file 1: Figure S1. The elution of the interacting proteins was loaded on 12% SDS-PAGE gel stained with silver nitrate and the bands were excised from gel and submitted to electroelution. The selected proteins were identified by MS–MS. A list of proteins found only associated to IN•viral DNA complexes but not to control DNA alone and linked to chromatin or transcription is provided here in addition to the factors reported in Table 1.

**Additional file 3: Figure S3.** *In vitro* interaction between HIV-1 IN, FACT and LEDGF/p75 variants. Immunoprecipitation or GST pull down were performed using recombinant cofactors, polyclonal anti-HIV-1 IN or anti-LEDGF antibodies. The interactions were monitored by direct gel staining using colloidal blue and quantified by Image J software.

**Additional file 4: Figure S4.** FAIRE analysis and effect of UV-crosslinking on chromatin FACT and integration activities. FAIRE analyses were performed as indicated in materials and methods section and schematized in (**A**, adapted from [54]). Analysis of nucleosome remodeling activity of FACT on chromatinized substrates in vitro was performed by quantifying the free DNA recovered after FAIRE assay performed on naked p5S or chromatinized p5S loaded on 1% agarose gel after treatment or not with FACT complex and UV-treated or untreated (**B**). The recovered DNA was then quantified using ImageJ software and data are shown as the mean ± standard deviation (error bars) of at least three independent sets of experiments (**C**). A typical concerted integration performed with DNA substrate pre-treated with UV before FACT addition is reported in (**D**). Analysis of nucleosome remodeling activity of FACT on chromatinized substrates in vitro in the presence of absence of LEDGF/p75 is reported in (**E**).

**Additional file 5: Figure S5.** Effect of FACT curaxin inhibitors on in vitro HIV-1 integration, cell viability and chromatin structure. The structure of CBL100 and CBL137 and their effect in typical concerted integration catalyzed by HIV-1 IN are shown in (**A**). The viability of the cells treated with curaxins was measured using a typical MTT assay and data are shown in (**B**). Effect of curaxin treatment on chromatin structure was analyzed by FAIR, as previously shown (**C**).

**Additional file 6: Figure S6.** Effect of FACT-mediated chromatin remodeling chemical promotion on early steps of HIV-1 LAI virus and derived lentiviral vectors. HeLaP4 and HEK293T cells were treated with CBLC137 curaxin (0.1 µM) 6 h before cell infection with LAI wild type virus or transduction with lentiviral vectors. The effect on LAI virus (**A**) and viral vector (**B**) reverse transcription and integration was evaluated by quantitative PCR performed on the different viral DNA populations at 0–48 h post-transduction. All values are shown as the mean ± standard deviation (error bars) of at least three independent sets of experiments done in duplicates.

**Additional file 7: Figure S7.** Effect of FACT knock down on early steps of HIV-1 derived lentiviral vectors in HEK293T cells. The early steps of replication of lentiviral vectors in cells knockdown for SSRP1 (see Fig. 7) were evaluated by quantification of the viral DNA populations at 0–48 h post-transduction using quantitative PCR (data obtained after a 20 nM siRNA treatment are reported here). All values are shown as the mean ± standard deviation (error bars) of four independent sets of experiments done in duplicates.
