# Supplementary figures and images for: Modulation of chromatin structure by the FACT histone chaperone complex regulates HIV-1 integration
Source: Retrovirology. 2017 Jul 28;14:39. doi: 10.1186/s12977-017-0363-4 (PMC5534098; doi:10.1186/s12977-017-0363-4)

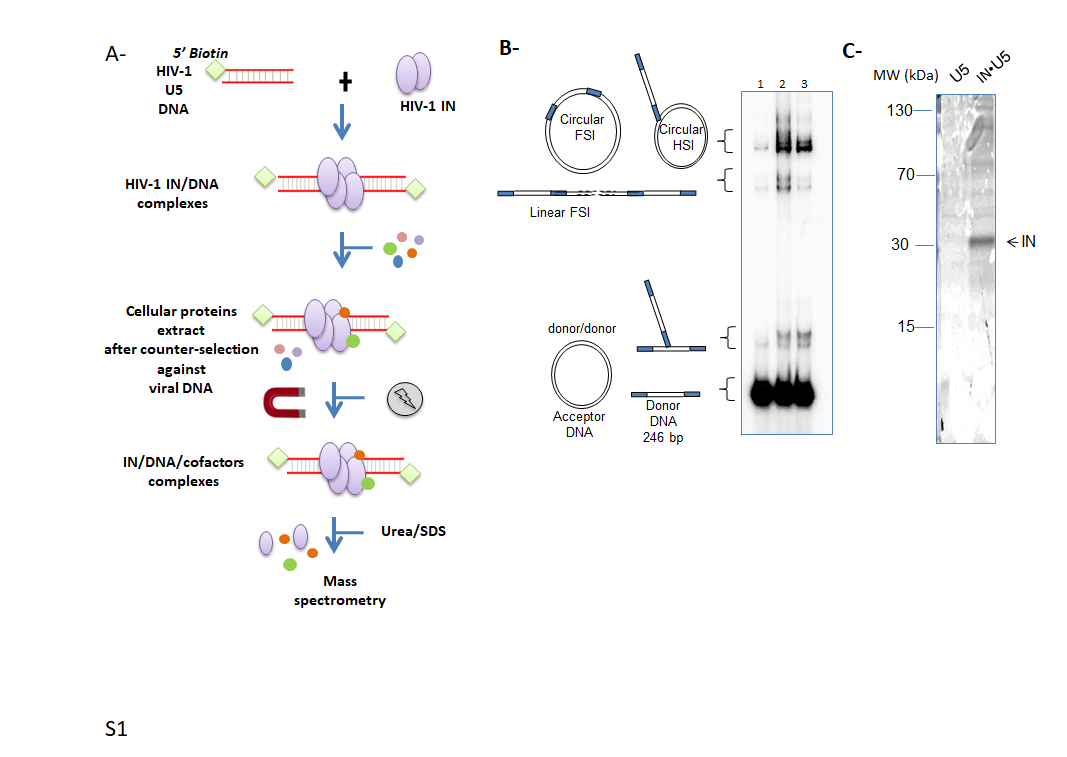

Supplement: Supplementary file 1 — Additional file 1: Figure S1. Strategy for selection of cellular interactants of IN•viral DNA complex. Cellular extracts from HeLa P4 cells were first incubated with streptavidin beads coupled to the viral DNA fragments in order to avoid selecting proteins binding solely to the DNA. The elution was then incubated with streptavidin beads coupled to fractions enriched in active IN•viral DNA complexes (A). Formation of active IN•DNA complexes was checked in vitro concerted integration (B), the data obtained with increasing concentration of INs, 100, 200, 400 nM (lanes 1–3) are reported. The elution of the interacting proteins was loaded on 12% SDS-PAGE gel stained with silver nitrate and the bands were excised and electroeluted. A typical result from a selection performed with the IN•viral DNA and the control DNA alone is reported in (C). [file 12977_2017_363_MOESM1_ESM.tif]

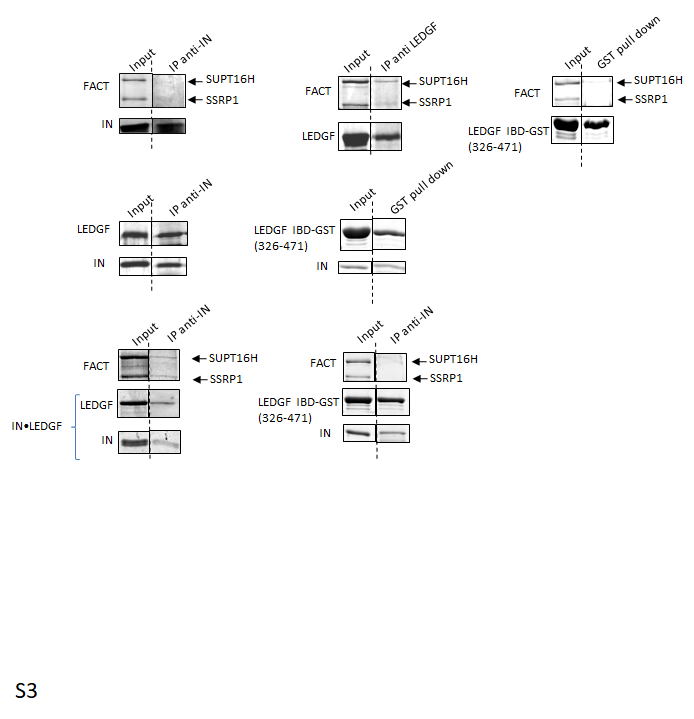

Supplement: Supplementary file 3 — Additional file 3: Figure S3. In vitro interaction between HIV-1 IN, FACT and LEDGF/p75 variants. Immunoprecipitation or GST pull down were performed using recombinant cofactors, polyclonal anti-HIV-1 IN or anti-LEDGF antibodies. The interactions were monitored by direct gel staining using colloidal blue and quantified by Image J software. [file 12977_2017_363_MOESM3_ESM.tif]

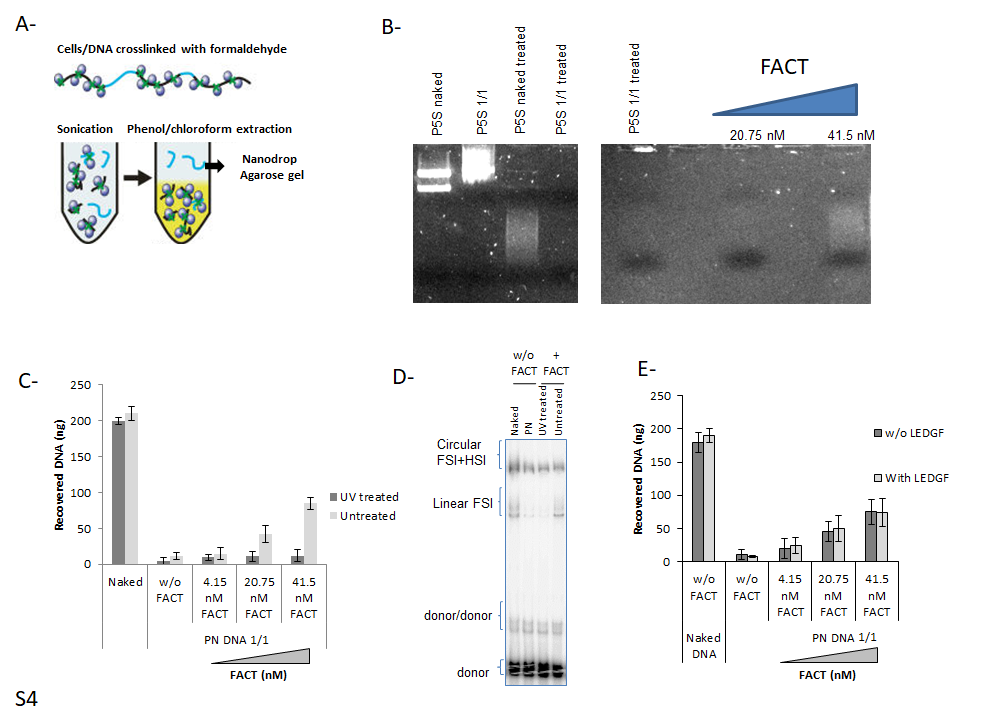

Supplement: Supplementary file 4 — Additional file 4: Figure S4. FAIRE analysis and effect of UV-crosslinking on chromatin FACT and integration activities. FAIRE analyses were performed as indicated in materials and methods section and schematized in (A, adapted from [54]). Analysis of nucleosome remodeling activity of FACT on chromatinized substrates in vitro was performed by quantifying the free DNA recovered after FAIRE assay performed on naked p5S or chromatinized p5S loaded on 1% agarose gel after treatment or not with FACT complex and UV-treated or untreated (B). The recovered DNA was then quantified using ImageJ software and data are shown as the mean ± standard deviation (error bars) of at least three independent sets of experiments (C). A typical concerted integration performed with DNA substrate pre-treated with UV before FACT addition is reported in (D). Analysis of nucleosome remodeling activity of FACT on chromatinized substrates in vitro in the presence of absence of LEDGF/p75 is reported in (E). [file 12977_2017_363_MOESM4_ESM.tif]

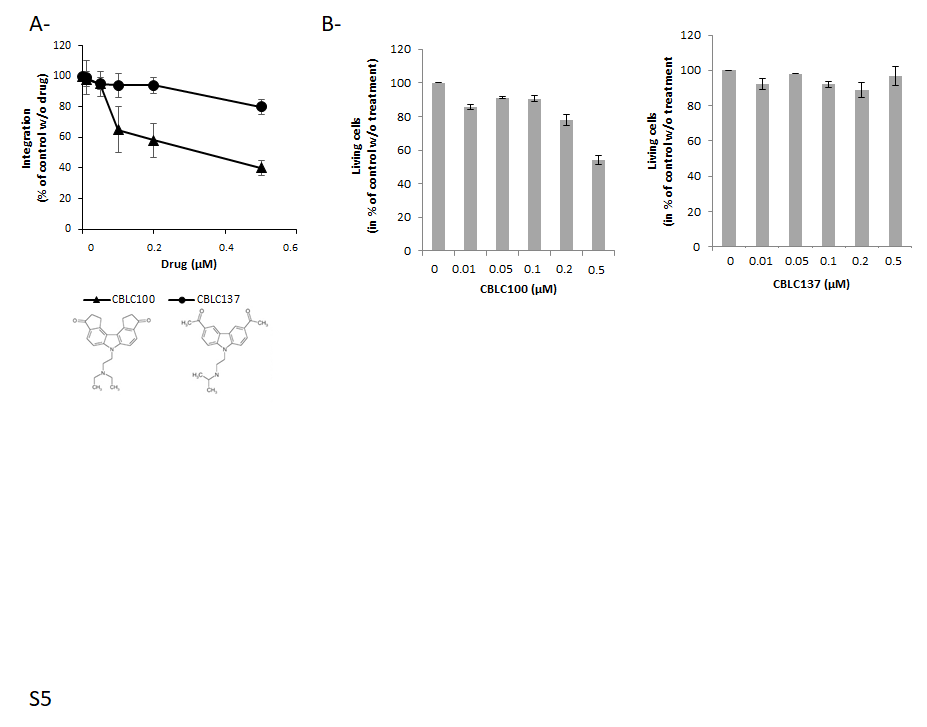

Supplement: Supplementary file 5 — Additional file 5: Figure S5. Effect of FACT curaxin inhibitors on in vitro HIV-1 integration, cell viability and chromatin structure. The structure of CBL100 and CBL137 and their effect in typical concerted integration catalyzed by HIV-1 IN are shown in (A). The viability of the cells treated with curaxins was measured using a typical MTT assay and data are shown in (B). Effect of curaxin treatment on chromatin structure was analyzed by FAIR, as previously shown (C). [file 12977_2017_363_MOESM5_ESM.tif]

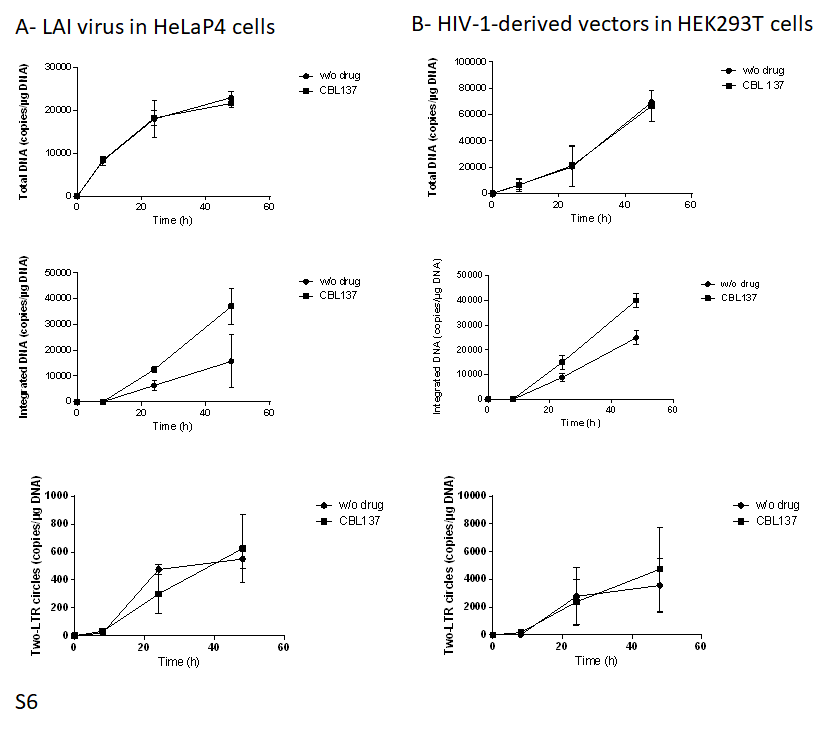

Supplement: Supplementary file 6 — Additional file 6: Figure S6. Effect of FACT-mediated chromatin remodeling chemical promotion on early steps of HIV-1 LAI virus and derived lentiviral vectors. HeLaP4 and HEK293T cells were treated with CBLC137 curaxin (0.1 µM) 6 h before cell infection with LAI wild type virus or transduction with lentiviral vectors. The effect on LAI virus (A) and viral vector (B) reverse transcription and integration was evaluated by quantitative PCR performed on the different viral DNA populations at 0–48 h post-transduction. All values are shown as the mean ± standard deviation (error bars) of at least three independent sets of experiments done in duplicates. [file 12977_2017_363_MOESM6_ESM.tif]

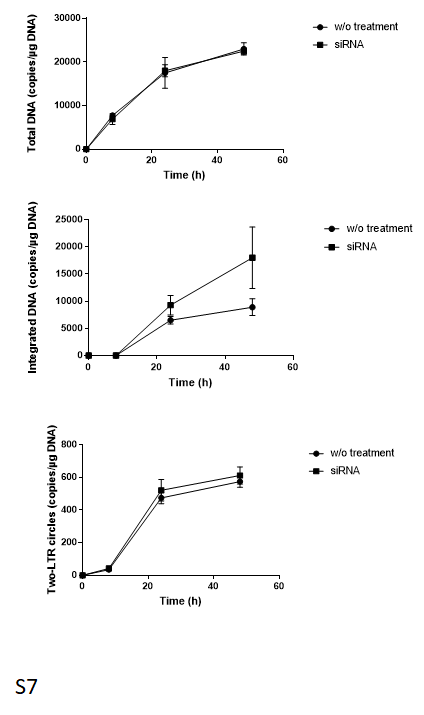

Supplement: Supplementary file 7 — Additional file 7: Figure S7. Effect of FACT knock down on early steps of HIV-1 derived lentiviral vectors in HEK293T cells. The early steps of replication of lentiviral vectors in cells knockdown for SSRP1 (see Fig. 7) were evaluated by quantification of the viral DNA populations at 0–48 h post-transduction using quantitative PCR (data obtained after a 20 nM siRNA treatment are reported here). All values are shown as the mean ± standard deviation (error bars) of four independent sets of experiments done in duplicates. [file 12977_2017_363_MOESM7_ESM.tif]
